# Tracheotomy in Croup

**Published:** 1860-09

**Authors:** C. S. Fenner

**Affiliations:** Memphis, Tennessee


ART. II.—*Tracheotomy in Croup.* Illustrated by Four Cases. By C. S. FENNER, M.D., of Memphis, Tennessee.

ON the 9th of November, 1857, I received a message from my friend, Dr. J. R. Frayser, requesting my presence at the residence of Mr. Borner, with a view to perform tracheotomy. Accompanied by Dr. W. D. Tucker, I immediately obeyed the summons, and found the patient, a little boy five years and three months old, who had been suffering for ten days with pseudo-membranous croup, in a dying condition. His lips were purple, pulse rapid and scarcely perceptible, and his respiration nearly suspended. I immediately cut through the integuments below the thyroid body, opened the trachea, and inserted a tube. The patient was entirely unconscious, evincing no signs of pain from the cutting. By the time the operation was completed, respiration having ceased, we resorted to artificial breathing, inflating the lungs through the tube for some ten or fifteen minutes, when he began to breathe through the instrument without assistance, and consciousness soon returned. The tube had to be taken out frequently and cleansed, as the orifice became plugged with mucus and detached pieces of membrane impeding or instantly checking respiration. At the end of thirty minutes he was so much revived that he called for water, and on being raised up drank freely. A few drops of a solution of nitrate of silver were occasionally poured through the opening into the trachea, which excited forcible expiration or coughing, causing the discharge of a quantity of tenacious mucus and flakes of false membrane through the tube, or, when that was removed, through the wound; after which he would breathe more easily and fall asleep. A small sponge probang was also repeatedly dipped in the nitrate of silver solution, and carried through the wound down to the bronchial tubes, and upward through the larynx On removing the probang, the tracheal secretion, coagulated by contact with the solution, would adhere to the sponge and be removed, with great relief to the little sufferer. There was also a free secretion of reddish colored serum, which, passing down into the lungs, obstructed respiration. I had him placed on his side, and the foot of the bed slightly elevated, so that the secretion would gravitate to the tracheal opening, and be discharged without any effort on his part. He passed a comfortable night, awakening only when the tube became obstructed, and dropping to sleep as soon as the obstruction was removed. On the eighth day the canula was removed and breathing went on through the wound until the tenth, when the opening closed and respiration was naturally and easily carried on through the larynx. He continued to improve in strength, and was regarded as nearly well. On the eighteenth day, while romping with some of his little playfellows, soon after eating a large piece of plum-cake, he was attacked with convulsions, and immediately became paralyzed on his entire right side, accompanied with symptoms indicating cerebral pressure. He lingered two days, and died the twentieth day from the operation. Up to the moment of death his breathing remained free and easy; and so far as the croup was concerned, the operation may be considered a successful one.

CASE 2.—On the 18th of January, 1858, I was called very early in the morning, by Dr. Frayser, to open the trachea of a little girl six years old, who was in the last stage of pseudo-membranous croup. As it was evident the patient could survive but a very short time, I inserted a canula in the trachea below the thyroid gland. Professional business calling me to an adjoining State, I did not remain with the patient, but Drs. Frayser and Chase attended her during the day and used every effort to keep up respiration, but without avail. She died fourteen hours after the operation.

CASE 3.—In August, 1858, Dr. Richard Taylor requested me to perform the operation of tracheotomy on a patient of his, an Irish girl five years of age. On visiting her, in company with Dr. W. V. Taylor, we found an extensive œdematous swelling in the cellular tissue of the neck over the trachea, and on cutting down found the trachea at least three inches from the surface. It was opened and a tube inserted, but owing to the great tumefaction of the parts, it was difficult to keep the tube in place, and still more so to clear it of shreds of membrane and mucus, so that respiration could go on. The patient lived but six hours after the operation.

CASE 4.—November 18th, 1858, I was called on to open the trachea of a most interesting little girl three years of age, in the last stage of membranous croup. Her friends having been informed by Dr. White, the attending physician, of the uncertainty of the operation, and the slight hope resulting from it, were anxious to have the effort made.

Assisted by Drs. White and Rice, I inserted the tube, and for twenty-four hours we had considerable hopes of her recovery, when respiration, which remained free, gradually increased in frequency until it became very rapid, evidently from the extension of inflammation to the smaller bronchial tubes, and she died forty-two hours after the operation.

CASE 5.—This case, although not one of membranous croup, still, as it was attended with croupal symptoms, I report it here:—

At one o'clock, Tuesday morning, April 3d, 1860, I was requested by my friends, Drs. Grant and Allen, to meet them in consultation at the residence of Mr. Simon. In a few minutes I was with them, and found their patient, Flora, a little daughter of Mr. Simon, three and a half years old, suffering from most painful dyspnœa, with croupal cough and entire loss of voice. From Dr. Grant I learned that the disease began about a week previous, with sore throat, enlarged and ulcerated tonsils, with some febrile action; but under the treatment adopted, she was thought to be improving until the day before, when a croupy cough came on, with great difficulty of breathing, which symptoms were constantly growing worse.

At eleven o'clock I again saw the patient, with her attending physicians, and found her nearly insensible, skin livid, pulse rapid, with considerable swelling of the throat externally, below the angle of the jaws; an anxious expression of countenance, and a struggling for breath painful to behold. Believing tracheotomy still offered a chance for life, we advised it; and Dr. Grant having obtained the consent of the parents, I inserted a tube in the trachea which gave immediate relief. She soon fell asleep, color and warmth returned to the extremities, and no unfavorable symptom supervened. For the first few hours the discharge from the tube was similar to that usually met with in membranous croup, after which the character of the secretion changed to a tenacious straw-colored mucus. On the morning of the seventh of April, she began to breathe a little through the nose, and on the eighth we removed the tube, it having been worn four days and eighteen hours. She breathed partially through the wound two days, when it closed, and respiration went on naturally without difficulty.

*Remarks.*—In France, tracheotomy in croup has been resorted to a great number of times, with a degree of success varying much with different operators. In the *Journal des Connaiss Med.*, June, 1839, we find the following statistical report:—

| | Operations. | Cures. | Deaths. |
|---|---|---|---|
| M. Amussat | 6 | 0 | 6 |
| M. Baudelocque | 15 | 0 | 15 |
| M. Blondin | 5 | 0 | 5 |
| M. Brettoneau | 18 | 4 | 14 |
| M. Gerdy | 6 | 4 | 2 |
| M. Roux | 4 | 0 | 4 |
| M. Trousseau | 80 | 20 | 60 |
| M. Velpeau | 6 | 0 | 6 |
| | 140 | 28 | 112 |

Here are thirty-six cases operated on by five surgeons who ranked among the ablest operators of France, all of which proved fatal, while in one hundred and four other cases in which the operation was performed by three other surgeons, twenty-eight were successful.

M. André, one of the internes of the Hôpital des Enfans, published an account of the operations of tracheotomy, in croup, performed in that institution during the year 1856, as shown in the following table:—

| Age. | Total. | Deaths. | | Recoveries. | |
|---|---|---|---|---|---|
| | | Boys. | Girls. | Boys. | Girls. |
| 15 months to 2 years........ ......... | 6 | 2 | 4 | ... | ... |
| 2 to 3 years........................... | 9 | 4 | 3 | 2 | ... |
| 3 to 4 years........................... | 13 | 5 | 4 | 4 | ... |
| 4 to 5 years........................... | 11 | 6 | 3 | 1 | 1 |
| 5 to 6 years........................... | 6 | 3 | 1 | 1 | 1 |
| 6 to $6\frac{1}{4}$ years.......................... | 3 | 1 | 1 | ... | 1 |
| 7 years................................. | 2 | ... | 1 | ... | 1 |
| 8 years................................. | 2 | ... | 1 | 1 | ... |
| 9 years................................. | 1 | ... | ... | 1 | ... |
| $9\frac{1}{2}$ years................................. | 1 | ... | ... | ... | 1 |
| | 54 | 21 | 18 | 10 | 5 |

The success of M. Trousseau and other operators has induced many to regard croup, as it occurs in France, as a diphtheritic form of the disease, differing from the inflammatory croup which is generally met with in this country. The former is principally confined to the throat and larynx; hence it is regarded as more favorable for an operation than the latter, which seems to poison the system, so that, even if the air has free access to the lungs, the disease still extends from the trachea to the bronchial tubes, and to the substance of the lungs, terminating in pneumonia and death.

In January, 1857, Dr. Fuller read to the Royal Medico-Chirurgical Society, a paper on tracheotomy in croup, a synopsis of which is given in the *Med. Times and Gazette*, February 7, 1857. In regard to the supposition that there is a difference in the disease in the two countries, the reporter says: "The fallacy of this supposition, he then proceeds to demonstrate by reference to the writings of French authors, and to the recorded results of the post-mortem investigation of 311 fatal cases of croup in France; and he showed that in regard to its pathological effects, diphtheritis, when accompanied by croupal symptoms, does not, as compared with inflammatory croup, present any greater prospect of success for the operation than it does in the character of its accompanying fever or the condition of the throat externally."

Dr. West was placed by Dr. Fuller among those who opposed the operation. "Dr. West observed that he must beg to set Dr. Fuller right with reference to the opinions which he, Dr. West, had expressed, in the published lectures concerning the operation of tracheotomy. So far from being an opponent of it, he had ventured to dissent from authorities high as Dr. Cheyne and Mr. Porter, and to advocate its adoption most decidedly. At the same time, his own personal experience of the operation, amounting to about ten cases, and that likewise obtained by his colleagues at the Children's Hospital, had not yet afforded a single instance of recovery. At the Children's Hospital every possible attention was paid to the circumstances in which the child was placed after the operation, while its performance was not delayed till the case was hopeless, but was performed comparatively early; and appropriate antiphlogistic treatment, including the employment of mercurials, was sedulously continued afterwards. He still believed that the difference in the character of the disease in England and France had much to do with the different results of tracheotomy in the two countries; and his own experience was that the cases of croup in this country, in which the affection of the larynx was unattended either by bronchitis or pneumonia, were a minority, and a very small minority, of the total number."

The numerous cures in France may possibly be accounted for by bearing in mind the fact that the operation is there generally performed in the second stage of the disease, or as soon as effusion of lymph begins to take place about the fauces. Every physician who has practiced any considerable length of time has seen cases of croup recover, from the efforts of nature, or from the effects of medicinal agents, even when the false membrane extended far down into the larynx. I myself, a few months since, saw the case of a little boy, six years of age, who had been gradually growing worse from croup for ten days; a false membrane covered the fauces and extended to the trachea; respiration was exceedingly laborious, his face livid and swollen, and when I saw him he was insensible. I was urgently solicited by his parents, and others around his bed, to open the trachea as the only means of averting what they considered certain dissolution, but I was unwilling to perform the operation so long as there was any effort at respiration. The little fellow gradually revived and finally recovered, although it was many weeks before he regained his voice. Had I opened the trachea, from the condition he was in, I should have most certainly attributed his recovery entirely to the operation, and should have regarded it as a successful case of tracheotomy in croup. Doubtless many cases have been operated on that would have recovered if left alone, and the cure attributed to the operation. In a recent discussion at the Academy of Medicine, a distinguished member stated that in France, within the last few years, the mortality of croup had considerably increased, and he attributed this increase to the great number of operations which had been performed, thus adding to the dangers of the disease the dangers of the operation. If tracheotomy is performed early, before the urgency of the symptoms imperatively demand it, and the patient recovers, it is by no means certain that the recovery is due to the operation; hence the statistics of French surgeons, who advocate this treatment, are not considered by the profession in other countries as very conclusive in determining the value of the operation.

My own opinion is, that it should never be performed except as a *dernier ressort*, when every other remedy has failed and death seems inevitable.

